# Control of protein degradation by N-terminal acetylation and the N-end rule pathway

**DOI:** 10.1038/s12276-018-0097-y

**Published:** 2018-07-27

**Authors:** Kha The Nguyen, Sang-Hyeon Mun, Chang-Seok Lee, Cheol-Sang Hwang

**Affiliations:** Department of Life Sciences, Pohang University of Science and Technology, Pohang, Gyeongbuk 37673 Republic of Korea

**Keywords:** Protein folding, Protein folding

## Abstract

Nα-terminal acetylation (Nt-acetylation) occurs very frequently and is found in most proteins in eukaryotes. Despite the pervasiveness and universality of Nt-acetylation, its general functions in terms of physiological outcomes remain largely elusive. However, several recent studies have revealed that Nt-acetylation has a significant impact on protein stability, activity, folding patterns, cellular localization, etc. In addition, Nt-acetylation marks specific proteins for degradation by a branch of the N-end rule pathway, a subset of the ubiquitin-mediated proteolytic system. The N-end rule associates a protein’s in vivo half-life with its N-terminal residue or modifications on its N-terminus. This review provides a current understanding of intracellular proteolysis control by Nt-acetylation and the N-end rule pathway.

## Protein Nα-terminal acetylation: Regulating stability

The addition of an acetyl group to amino acids at the start of a protein (known as the Nα-terminal end) is crucial for maintaining protein homeostasis and cellular health. Hwang and colleagues review the effects of Nα-terminal acetylation (Nt-acetylation) on protein function and stability. This modification occurs in over 50% of proteins in eukaryotic organisms and when mis-regulated can lead to cancer, hypertension, neurodegeneration etc. Nt-acetylation not only targets proteins for degradation through a specific signaling pathway, but also regulates protein folding, cellular localization and activity. This seemingly irreversible modification can also protect proteins against other mechanisms of degradation and represents a potential therapeutic target. Inhibiting Nt-acetylation-dependent protein interactions could be a useful strategy for regulating protein stability.

## Introduction

The steady-state levels of intracellular proteins and their integrity are orchestrated according to the physiological demands. Consequent protein homeostasis is central to fundamental biological processes, cellular and organismal health, and lifespan^1,2^. To maintain and regulate protein homeostasis, cells have developed highly robust mechanisms that include protein refolding, sequestration, spatial compartmentation, and degradation^1,2^. The failure of cellular quality control in protein homeostasis causes many devastating maladies such as neurodegenerative disorders, cancers, and autoimmune diseases^1,2^.

**Fig. 1:**
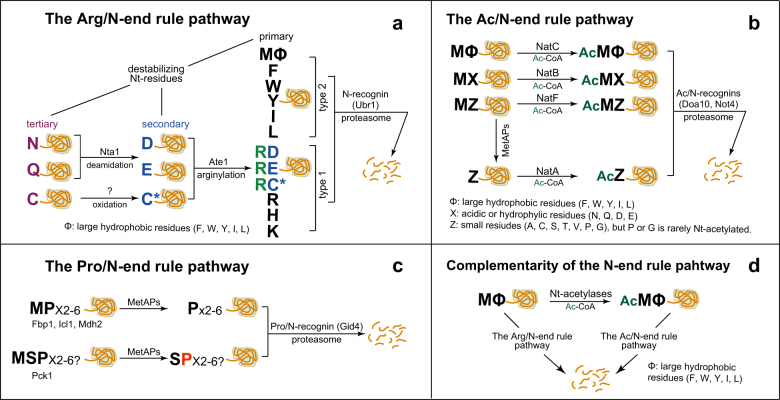
The N-end rule pathway in *S. cerevisiae*. **a** The conventional Arg/N-end rule pathway. Nta1 N-terminal amidase deamidates tertiary destabilizing N-terminal residues Asn and Gln into secondary destabilizing N-terminal residues Asp and Glu, respectively. Subsequently, Ate1 arginyl-transferase attaches Arg to the N-terminal Asp or Glu. Eventually, Ubr1 recognizes the type-1 or type-2 primary destabilizing N-terminal residues of the target substrates for polyubiquitylation and subsequent proteasomal degradation. Note that the N-terminal Cys oxidation-dependent degradation pathway has not yet been established in yeast. **b** The Ac/N-end rule pathway. Met aminopeptidases (MetAPs) remove the N-terminal initiator Met (iMet), if the residues smaller than Val occupy the penultimate position. NatA, NatB, or NatC Nt-acetylates target proteins largely based on their first two residues. The Ac/N-recognins (Doa10 and Not4) specifically recognize the N-terminal acetyl moiety of the N-terminally acetylated proteins for polyubiquitin-mediated and proteasome-dependent degradation. **c** The Pro/N-end rule pathway. N-terminal Pro or N-terminal Ser with Pro at position 2 can work as Pro/N-degrons for Pro/N-recognin Gid4 in the GID Ub ligase complex. **d** Functional and mechanical interplay between the two branches of the N-end rule pathway

Regulated degradation of specific proteins is performed mostly by the ubiquitin (Ub)-proteasome-system (UPS). UPS recruits the cascade of reactions consisting of Ub-activating E1s, Ub-conjugating E2s, and Ub ligase E3s, and sequentially and covalently attaches Ub to the target proteins^3,4^. In particular, E3s specifically recognize the structural features or sequence contexts of target substrates termed degradation signals (degrons), thus polyubiquitylating them for proteolysis with the 26S proteasome^3,4^. Interestingly, N-terminal residues or their modifications have been established as specific degrons (termed N-degrons) by the N-end rule pathway, a part of the UPS, over the past 30 years (Fig. 1)^4–11^.

Nα-terminal acetylation (Nt-acetylation) is a highly prevalent modification affecting ~50–80% of the cellular proteins in eukaryotes^12,13^. Nt-acetylation contributes to a broad range of cellular processes including cell proliferation, apoptosis, development, stress responses, and immune regulation^12–20^. Additionally, dysregulation of Nt-acetylation results in many severe pathological conditions such as cancers, X-linked genetic disorders, hypertension, and neurodegenerative diseases^13,21–27^. At the molecular level, Nt-acetylation of specific proteins significantly influences their stability, activity, folding, and localization^25,28–33^. Furthermore, Nt-acetylation triggers the degradation of specific proteins by a branch of the N-end rule pathway, termed the Ac/N-end rule pathway^4,14,34^.

Here, we will briefly review our current understanding of how Nt-acetylation and the N-end rule pathway could control intracellular proteolysis, thus affecting protein homeostasis.

## The specificity of Nt-acetylation

Nt-acetylation is carried out by specific Nt-acetylases that catalyze the attachment of an acetyl moiety from acetyl-CoA to the N-termini of the cellular proteins. Nt-acetylases are grouped into NatA, NatB, NatC, NatD, NatE, NatF, and NatG complexes according to their substrate specificity, subunit compositions, and subcellular compartmentation^13^. NatA–NatE are conserved from yeast to humans, whereas Golgi-attached NatF and chloroplast-resident NatG are found exclusively in multicellular eukaryotes and plants, respectively^13,35–37^.

The substrate specificity of Nt-acetylases is determined largely by the first two residues at the N-terminus^13^. For example, NatA acetylates N-terminal Ala, Ser, Cys, Val, Thr, or Gly, which is largely generated by the excision of initiator Met (iMet) via Met-aminopeptidases (MetAPs). NatD also works on iMet-processed N-termini, but it solely acetylates the N-terminal residues of histones H2A and H4^13^. On the other hand, NatC, NatE, and NatF acetylate N-terminal Met. In particular, NatB works on N-terminal Met bearing Asp, Glu, Asn, or Gln at position 2, whereas NatC, NatE, and NatF work on Met-Leu-, Met-Ile-, Met-Tyr-, or Met-Lys-starting residues. However, chloroplast-specific NatG promiscuously acetylates N-terminal Met, Ala, Ser, or Thr^13^.

## Three branches of the eukaryotic N-end rule pathway

Specific N-terminal residues or their modifications comprise N-degrons. The recognition components of N-degrons and their associated proteolytic system are called N-recognins (usually E3 Ub ligases in eukaryotes) and the N-end rule pathway, respectively^4–11^. The N-end rule pathway enables N-degrons to dictate the in vivo half-life of a protein^4–11^.

In eukaryotes, three different branches of the N-end rule pathway are hitherto established: the Arg/N-end rule pathway, the Ac/N-end rule pathway, and the Pro/N-end rule pathway (Fig. 1)^38,39^.

In the Arg/N-end rule pathway, the dedicated N-degrons include N-terminal Arg, Lys, His, Leu, Trp, Phe, Ile, Tyr, or Met-Φ (Met with a hydrophobic residue at position 2)^4–10,40^. In addition, N-terminal Asn or Gln can become destabilizing residues after their deamidation into Asp or Glu and subsequent N-terminal arginylation (Fig. 1a). Ultimately, N-recognins such as Ubr1 in *Saccharomyces cerevisiae*, UBR family E3 ligases (UBR1, UBR2, UBR4, UBR5) in mammals, or PRT1 and PRT6 in plants directly target the unmodified destabilizing N-terminal residues for polyubiquitylation-mediated degradation by the 26S proteasome (Fig. 1a)^4–10,40,41^. In multicellular eukaryotes, the N-terminal Cys can be oxidized by oxygen, nitric oxide, or Cys oxidases and subsequently arginylated^42–45^. Interestingly, an autophagy receptor SQSTM1 (p62) also acts as an N-recognin of the Arg/N-end rule pathway by directly recognizing the destabilizing N-terminal residues of aggregated proteins for autophagy-dependent degradation, although it is not an E3 Ub ligase^46,47^. The Arg/N-end rule pathway functions in protein quality control, small peptide sensing, apoptosis, neurodegeneration, DNA repair, G-protein signaling, plant development, etc^4–11^.

Another branch of the N-end rule pathway is the Ac/N-end rule pathway, which conducts the degradation of N-terminally acetylated proteins by directly targeting their N-terminal acetyl moiety (Fig. 1b)^14,34^. The recognition component and the degradation signal of the Ac/N-end rule pathway are termed as Ac/N-recognin and Ac/N-degron, respectively^14,34^. Ac/N-recognin includes the endoplasmic reticulum (ER) transmembrane Doa10 (its mammalian counterpart is TEB4) and cytosolic/nuclear Not4 E3 Ub ligases in *S. cerevisiae*^14,23,48,49^. The Ac/N-end rule pathway functions in the control of protein quality, subunit stoichiometry in complexes, blood pressure via G-protein signaling, circadian rhythm, and plant immunity and stress responses^14,16,40,48,50^.

The third branch of the N-end rule pathway is the Pro/N-end rule pathway, which recruits Gid4, a subunit of the oligomeric GID (glucose-induced degradation-deficient) E3 Ub ligase, as a Pro/N-recognin in *S. cerevisiae* (Fig. 1c)^38,39^. Gid4 specifically recognizes the N-terminal Pro or penultimate Pro in conjunction with the adjacent residues of its substrates. The Pro/N-end rule pathway conducts the degradation of a subset of gluconeogenic enzymes such as Fbp1 (a fructose-1,6-bisphosphatase), Icl1 (an isocitrate lyase), Mdh2 (a cytoplasmic malate dehydrogenase), or Pck1 (a phosphoenolpyruvate carboxykinase), depending upon the glucose availability in the growth media^38^. Despite the existence of the conserved GID Ub complex counterparts, the Pro/N-end rule pathway in mammals and plants remains to be elucidated.

## Complementarity of the branches of the N-end rule pathway

Despite the pervasiveness and abundance of Nt-acetylation, many proteins are partially N-terminally acetylated, and some proteins are very rarely N-terminally acetylated^12,13^. Moreover, the efficiency and the extent of Nt-acetylation could be determined by Nt-acetylases, target substrates, and acetyl-CoA availability, according to various cellular states^12,13,20^. Importantly, conditional regulation of Ac/N-degrons (through either steric shielding/unshielding or spatial compartmentation) suggests a putative proteolytic system that could monitor and thereby obliterate the N-terminally unacetylated proteins^34,48^. Indeed, the unacetylated N-terminal Met of nearly all nascent cellular proteins works as a specific destruction signal termed MΦ/N-degron, provided that a hydrophobic residue (Φ) occupies the penultimate position^40^. Detailed analysis of the MΦ/N-degrons made it possible to identify a new function of the Arg/N-end rule pathway in the removal of the N-terminally unacetylated MΦ-containing proteins and further revealed the functional and mechanical crosstalk between the Arg/N-end rule pathway and the Ac/N-end rule pathway (Fig. 1d)^40,49^. For instance, yeast Msn4 (a stress–response transcription factor), Sry1 (a 3-hydroxyaspartate dehydratase), Arl3 (a small GTPase of the RAS superfamily), Pre5 (a 20S proteasome subunit), human hypertensive ML-RGS2 (a G-protein signaling regulator 2 variant) bearing a Gln→Leu mutation at position 2, and rodent AANAT (a serotonin N-acetyltransferase) are eliminated by cooperation between the Arg/N-end rule pathway and the Ac/N-end rule pathway, depending upon their Nt-acetylation states^23,40,50^.

## Conditionality of Ac/N-degrons

**Fig. 2:**
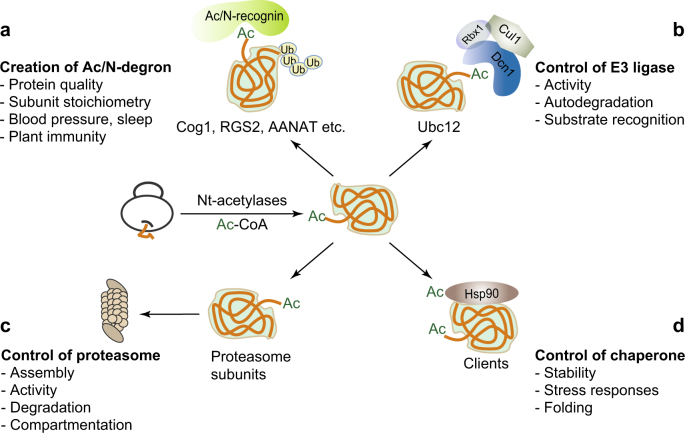
Control of protein degradation by Nt-acetylation. **a** N-terminally acetylated proteins can be targeted for degradation by the Ac/N-end rule pathway. **b** Nt-acetylation controls the activity or degradation of E3 ligases. **c** Nt-acetylation contributes to the activity, assembly, degradation, and compartmentation of 26S proteasomes. **d** Nt-acetylation protects Hsp90 and its clients from degradation. Unacetylated Hsp90 and its unacetylated clients are targeted for degradation by the Arg/N-end rule pathway

Nt-acetylation is a major abundant protein modification, and it takes place cotranslationally (post-translationally as well) and seemingly irreversibly^12,13,51^. Thus, the majority of cellular proteins most likely retain the Ac/N-degrons from the moment of their birth to death (Fig. 2a)^34,52^. In contrast to this hypothetical inference of pervasive built-in Ac/N-degrons, most Nt-acetylated proteins remain stable and long-lived. This apparent discrepancy could be explained by the steric shielding of Ac/N-degrons in normal or native proteins through rapid intramolecular folding and intermolecular sequestration by the binding partners or subcellular compartmentation^4,34,40,48,49^. Consequently, the steric shielding or sequestration of the Ac/N-degrons would protect the Nt-acetylated proteins from degradation by the Ac/N-end rule pathway. Indeed, several short-lived proteins (Cog1, Hcl1, and RGS2) become long-lived when their Ac/N-degrons are sterically shielded or become inaccessible upon coexpression with their binding partners Cog2-4, Cut9, or Gαq, respectively^23,48^. The Ac/N-end rule pathway therefore plays crucial roles in the control of protein quality and subunit remodeling or stoichiometry in complexes (Fig. 2a)^40,48^.

## Control of E3 ligases by Nt-acetylation

Nt-acetylation converts the positively charged α-amino group of the cellular proteins into a hydrophobic moiety, thus driving the interactions between the Nt-acetylated proteins and their binding partners^13^. For example, Nt-acetylation facilitates complex formation in anaphase-promoting/cyclosome (APC/C) subunits^53^, tropomyosin-actin^54^, Sir3 (a silence information regulator)-nucleosome^55,56^, and PLD3 (phsoducin-like 3)-VEGFR-2 (vascular endothelial growth factor 2)^57^. In particular, Schulman and colleagues found through structural and biochemical analyses that N-terminally acetylated Met of an E2 Ubc12 deeply docks into the hydrophobic cleft of Dcn1, a subunit of the Nedd8 E3 ligase complex, promoting neddylation to the target cullin proteins. As a result, the neddylated cullins stimulate the activity of cullin-RING ligases (Fig. 2b)^29,58^. Importantly, the Nt-acetylation-dependent interaction of the Dcn1 family with Ubc12 E2s is structurally and mechanistically conserved from yeast to mammals^59^. Most recently, they have developed small molecule inhibitors that specifically and potently block the Nt-acetylation-mediated binding of the Dcn1 family to Ubc12 E2s, and subsequently decrease the neddylation activity of the E2–E3 complex without perturbing global protein homeostasis. More importantly, small molecule inhibitors effectively suppress cancer cell growth in an anchorage-independent way, suggesting that specific recognition of the Nt-acetyl moiety could be a promising drug target^60^.

Although Doa10 and TEB4 E3 Ub ligases are the ER-embedded Ac/N-recognins in *S. cerevisiae* and mammals, respectively, their regulation with respect to the Ac/N-end rule pathway is poorly understood to date. Similar to other E3 Ub ligases, TEB4 restricts its expression levels via autoubiquitylation and subsequent proteasomal degradation^61^. Interestingly, short-lived TEB4 bearing N-terminal Met-Asp is N-terminally acetylated by NatB, suggesting its possible degradation via the Ac/N-end rule pathway (Fig. 2b)^62^. On the other hand, TEB4 mediates the degradation of squalene monooxygenase, one of the rate-limiting enzymes in cholesterol biosynthesis^63,64^. All the carbon atoms in the cholesterol stem from acetyl-CoA, a co-substrate of Nt-acetylation. Moreover, the intracellular level of acetyl-CoA is subject to metabolic control and acts as a rate-limiting factor for internal Nε-Lys acetylation^65^. Hence, acetyl-CoA availability would also most likely influence the efficiency or extent of Nt-acetylation. Indeed, Yi et al. showed that overexpression of anti-apoptotic protein Bcl-xL leads to hypo-Nt-acetylation by lowering intracellular acetyl-CoA levels, thus linking acetyl-CoA availability to Nt-acetylation of intracellular proteins upon apoptosis^20^. Taken together, TEB4 acts as a key player of a specific feedback circuit in the Ac/N-end rule pathway by sensing acetyl-CoA availability for the degradation of TEB4 itself in response to environmental or metabolic changes.

On the other hand, the E3 Ub ligases of the Arg/N-end rule pathway directly recognize the α-amino group of the target proteins, which could be potentially blocked by Nt-acetylation. In addition, Nt-acetylation impedes the attachment of Ub to the α-amino group of Met by linear Ub chain assembly complex (LUBAC), E3 ligase^66,67^ or Ub-conjugating Ube2W E2 enzyme^68^. Thus, the competitive interplay between Nt-acetylation and ubiquitylation provides another regulatory mechanism for protein stability or signaling.

## Control of the 26S proteasome by Nt-acetylation

The 26S proteasome comprises two 19S regulator particles (RPs) and a single 20S core particle (CP). Among the 35 subunits of the *S. cerevisiae* 26S proteasome, 21 are N-terminally acetylated^69–74^. Interestingly, the latent 20S CP of *naa10∆* cells (lacking the catalytic subunit of NatA Nt-acetylase) retained slightly higher chymotrypsin-like activity than the wild-type cells^69,71^. Thus, Nt-acetylation appears to affect the assembly of the 20S CP by tightly closing its channel in the latent state. In fact, crystallographic analysis revealed that the N-terminal region of α-subunits closes the channel of the latent CP. Moreover, ablation of the N-terminal nine residues of the α3 subunit slightly augments the chymotrypsin-like activity of the CP. Hence, Nt-acetylation of the α-subunits appears to influence the closing step in the gated channel of the CP^75^. However, purified proteasome holoenzyme (26S proteasome) shows no differences in the proteolytic activity toward its model substrates^72^. Therefore, it still remains to be verified whether Nt-acetylation of CP and RP subunits can control the proteasome activity directly.

On the other hand, out of the seven β subunits of the 20S CP in *S. cerevisiae*, the proteolytic subunits β1 (Pre3), β2 (Pup1), and β5 (Doa3) contain N-terminal propolypeptides, which are autocatalytically removed during proteasome assembly^76–78^. Consequently, the α-amino group of the exposed N-terminal Thr acts as a catalytic nucleophile during substrate hydrolysis, forming proteolytically active β subunits. Interestingly, the N-terminal propolypeptides seem to prevent Nt-acetylation of the N-terminal catalytic Thr residue, as engineered β1, β2, and β5 subunits lacking Nt-propolypeptides significantly decrease the protease activity of the 26S proteasome through Nt-acetylation of their exposed Thr residue^77^. In addition to the assembly and activity of 26S proteasomes, Nt-acetylation affects their localization. In *S. cerevisiae*, 26S proteasomes mainly reside in the nucleus under nutrient-rich conditions, but they are re-localized to cytosol in response to nutrient depletion, thus forming cytoplasmic aggregate-like structures called proteasome-storage granules (PSGs). Interestingly, the absence of either NatB or NatC Nt-acetylases abolishes the re-localization of 26S proteasomes during nutrient starvation, although it is unclear how NatB or NatC modulates the nucleus-to-cytosol distribution of the 26S proteasome^79^.

Furthermore, Nt-acetylation impedes the degradation of the 20S CP subunit α6 (Pre5) by the Arg/N-end rule pathway in *S. cerevisiae*. Pre5 starts with N-terminal Met-Phe and contains MΦ/N-degron. Pre5 is relatively stable in the wild-type, *ubr1Δ* (Arg/N-end rule pathway-lacking), or *naa30Δ ubr1Δ* (both NatC Nt-acetylase catalytic subunit and Arg/N-end rule pathway-lacking) cells, but it becomes destabilized in *naa30Δ* (NatC Nt-acetylase catalytic subunit-lacking) cells. Remarkably, the slow growth of *naa30Δ* cells at 37 °C could be partially rescued by Pre5 overexpression or by further ablation of Ubr1. Therefore, Nt-acetylation most likely blocks the recognition of Ubr1 by the MΦ/N-degron of Pre5, thereby preventing the degradation of Pre5 by the Arg/N-end rule pathway^40^. Collectively, Nt-acetylation controls numerous aspects of the 26S proteasome including assembly, remodeling, activity, stability, and localization for physiological needs (Fig. 2c).

## Control of molecular chaperones by Nt-acetylation

Molecular chaperons assist in protein folding/unfolding, quality control, disaggregation, stabilization, degradation, etc^1,2^. Since the attachment of an acetyl moiety to the α-amino group alters the local structures and chemical properties, Nt-acetylation seems to regulate the expression and activity of molecular chaperones, thus making it possible to maintain protein homeostasis and its combinatorial cellular phenotypes. In line with this conjecture, loss of NatA Nt-acetylase results in the accumulation of aberrant proteins, subsequently augments the expression levels of the molecular chaperons, and reassigns these chaperons to relive the proteotoxic stresses, thereby repressing the filamentous aggregation of yeast Sup35/PSI + prion^80^. Additionally, the absence of Nt-acetylation causes the aggregation of neuropathological proteins such as huntingtin and α-synuclein^24,25,81^. Furthermore, the molecular chaperons can directly modulate the Nt-acetylase activity. For instance, a chaperone-like protein HypK (Huntingtin yeast two-hybrid protein K) tightly interacts with the human NatA complex, thus contributing to its activity^81^.

Most recently, Varshavsky and colleagues found that Nt-acetylation and the N-end rule pathway control the Hsp90 chaperone system in *S. cerevisiae* (Fig. 2d)^82^. Interestingly, Hsp90 and their cochaperones are identified or predicted to be NatA substrates. Remarkably, the loss of Naa10 greatly deteriorates the Hsp90 chaperone system and thereby significantly reduces the binding affinity to its clients including Chk1 (a mitotic check point kinase). Consequently, the released N-terminally unacetylated Chk1 is quickly degraded by the Arg/N-end rule pathway, and N-recognin Ubr1 E3 ligase, a component of this pathway, directly recognizes the exposed internal degron at the C-terminus-proximity region of Chk1. Upon the ablation of Naa10, the Arg/N-end rule pathway also targets Hsc82 (the main component of Hsp90) and its clients such as Kar4 (a pheromone-response transcription regulator), Tup1 (a transcription co-repressor), Gpd1 (glycerol-3-phosphate dehydrogenase), or Ste11 (a MEK kinase) for degradation. More interestingly, the absence of Naa10 results in a ~2.5-fold increase in the expression levels of Ubr1, and strongly upregulates the Arg/N-end rule pathway, suggesting specific regulatory circuits that could operate across all eukaryotic species due to evolutionarily conserved Nt-acetylases, the chaperone system, and the N-end rule pathway^82^.

## Concluding remarks

Protein homeostasis is a pivotal life process, and failure of its maintenance causes numerous proteotoxic stresses leading to intractable diseases^1,2^. Recent works have revealed the crucial functions of Nt-acetylation in protein homeostasis, for example, Nt-acetylation not only acts as Ac/N-degrons of the N-end rule pathway, but also modulates the activity, assembly, or compartmentation of E3 ligases, 26S proteasomes, and chaperones (Fig. 2). Nonetheless, many key questions about the effects of Nt-acetylation, particularly on the protein degradation, remain to be addressed: (1) How do environmental or metabolic changes control the Nt-acetylated proteome? (2) What are the underlying molecular mechanisms and structural prerequisites for Ac/N-degron recognition? (3) Could Nt-acetylated small metabolites regulate the Ac/N-end rule pathway? (4) What are the physiological functions and pathological phenotypes of the Ac/N-end rule pathway in multicellular organisms? (5) Could the Ac/N-end rule pathway represent therapeutically applicable targets for the treatment of proteotoxic or metabolic diseases?
